# Aqueous microwave-assisted cross-coupling reactions applied to unprotected nucleosides

**DOI:** 10.3389/fchem.2015.00010

**Published:** 2015-02-17

**Authors:** Gwénaëlle Hervé, Christophe Len

**Affiliations:** ^1^Sorbonne Universités, Université de Technologie de Compiègne, Ecole Supérieure de Chimie Organique et Minérale, EA 4297 Transformations Intégrées de la Matière Renouvelable, Centre de Recherche Royallieu Compiégne, France; ^2^Department of Chemistry, University of Hull Hull, England

**Keywords:** nucleoside, nucleotide, Suzuki-Miyaura, Heck, water, green chemistry, microwave

## Abstract

Metal catalyzed cross-coupling reactions have been the preferred tools to access to modified nucleosides (on the C5-position of pyrimidines and on the C7- or C8-positions of purines). Our objective is to focus this mini-review on the Suzuki-Miyaura and on the Heck cross-couplings of nucleosides using microwave irradiations which is an alternative technology compatible with green chemistry and sustainable development

## Introduction

Nucleoside, nucleotide and oligonucleotide analogs exhibit, for some of them, antiviral and/or antitumoral activities and can be used as synthetic probes for biomedical applications. Numerous modifications have been made on C5- and C6-positions of pyrimidines as well as on C7-position of deazapurines and C8-position of purines. Very recently, literature even refers to modifications directly on oligonucleotides. In order to furnish greener solutions for chemical and pharmaceutical companies, the synthesis of nucleoside analogs *via* cross-coupling reactions has widely been studied. Cross-coupling reactions have mainly been developed in the presence of palladium-based catalysts *via* the Suzuki-Miyaura, the Heck and the Sonogashira reactions (Agrofoglio et al., 2003). Among the 12 principles of green chemistry, the use of benign solvents (water) and auxiliaries, the design of energy efficiency (microwave) and the use of catalysis were the most reported. In the particular domain of nucleoside chemistry, only the Suzuki-Miyaura and Heck cross-coupling reactions were described in sole water under microwave irradiations.

## Microwaves assisted suzuki-miyaura cross-couplings in pure water

**Scheme 1:**
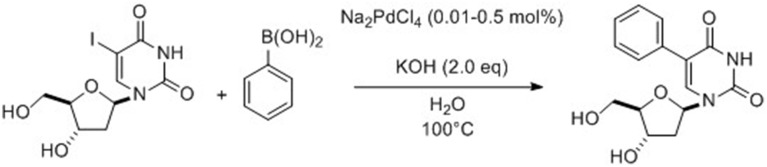
**Synthesis of 5-phenyl-2′-deoxyuridine from 5-iodo analog (Gallagher-Duval et al., 2013)**.

Fresneau et al. (2012) reported a Suzuki-Miyaura reaction starting from 2′-deoxyuridine in a completely aqueous medium using an inexpensive catalyst/ligand system which is also easily available. Model reaction conditions involved unprotected 5-iodo-2′-deoxyuridine and 4-methoxyphenylboronic acid. The best conditions consisted in the coupling of the unprotected chosen nucleoside in the presence of Na_2_CO_3_ (1.5 eq), Pd(OAc)_2_ (3 mol%) and PPh_3_ (5.4 mol%) in neat water at 80°C for few hours. In the course of their study, Fresneau et al. (2012) decided to consider, under their above described conditions, the influence of microwave irradiation at 120°C. This change in heating mode permitted a significant reduction in the reaction time (10 min vs. 4 h) while keeping the same efficiency. Application of this new method to different arylboronic acids afforded 13 cross-coupling products in good yields with substrates that contained either activating or deactivating groups in the *para* and *meta* positions of the aromatic ring. The same year, Len's group reported an efficient ligand-free Suzuki-Miyaura cross-coupling reaction in pure water starting from free 5-iodo-2′-deoxyuridine (Gallagher-Duval et al., 2013). The catalyst system was composed of very low amounts of Na_2_PdCl_4_ (0.05–0.1 mol%) and KOH (2 eq) (Scheme 1).

Various substrates with electron donating groups (EDG) and electron withdrawing groups (EWG) in the *para* or *ortho* positions (including sterically demanding boronic acids and heteroboronic acids) were systematically examined under both classical heating and microwave irradiations and 14 cross-coupling nucleoside derivatives were isolated (Gallagher-Duval et al., 2013). The coupling reactions were always more effective when the microwave irradiations were used. Three heteroboronic acids were also tested but it was found that only thiophen-2-boronic acid was reactive enough to give, in modest yields, the desired cross-coupling product using either conventional heating or microwave irradiation. Furan-2-yl boronic acid was sufficiently reactive only using the alternative technology.

## Microwaves assisted heck cross-couplings in pure water

Aqueous Heck cross-couplings applied to classical substrates are well documented (Casalnuovo and Calabrese, 1990; Fihri and Len, 2010; Polshettiwar et al., 2010; Fihri et al., 2011). However, only few recent examples of this reaction performed on nucleosides in aqueous conditions have been reported and none of them used microwaves irradiation assistance.

**Scheme 2:**
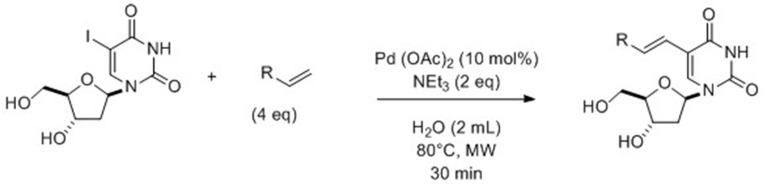
**Synthesis of 5-alkenyl-2′-deoxyuridine from 5-iodo analog (Hervé and Len, 2014)**.

In 2014, for the first time, Hervé and Len (2014) described a ligand-free Heck cross-coupling reaction under assisted-microwaves in pure water performed on 5-iodo-2′-deoxyuridine and various acrylate derivatives. Reactions were carried out using Pd(OAc)_2_ (10 mol%) with Et_3_N (2 eq) as base at 80°C (Scheme 2).

The reported method proceeded well with the acrylate having shortest chains since it permitted to provide the desired compounds in 90% yields. Reactions starting from more lipophilic esters were not so efficient and led to moderate yields (35–45%). The presence of a heteroatom (such as chlorine or oxygen) did not seem to change the hydrophobic/lipophilic balance of the reactant; consequently cross-coupling compounds were isolated in the same moderate range of yields. In order to have a large variety of compounds, substitution of the ester by amido and cyano groups was studied. Application of the methodology allowed access to the target compounds in respectively, 60 and 51% yields. The authors were able to synthesize the well-known antiviral BVDU in three successive totally aqueous steps: free-ligand microwaves-assisted Heck cross-coupling of 5-iodo-2′-deoxyuridine with methylacrylate; hydrolysis and Hunsdiecker reaction. Finally, this new methodology permitted to isolate BVDU with a better overall yield than that reported in the literature (56 vs. 31%) (De Clercq et al., 1986).

## Concluding remarks

Conventional syntheses of cross-coupling reactions for the preparation of new antiviral and antitumor nucleoside analogs have been well reported. During the last decade, the new synthetic objectives in this area were to design and to improve materials, products processes and systems. In this context, a challenging new avenue has involved the use of green solvents, catalysts, alternative technologies and energy efficiency. Few groups have already started to search in this domain leading to efficient green reactions. To date, among this scientific community, Len's group proposed the greenest process: efficient reactions starting from unprotected nucleosides with small loadings of palladium in the absence of ligand, in water, under microwave irradiations.

### Conflict of interest statement

The authors declare that the research was conducted in the absence of any commercial or financial relationships that could be construed as a potential conflict of interest. The author CL had previously collaborated with the reviewer RL.

## Correction note

A correction has been made to this article. Details can be found at: 10.3389/fchem.2026.1827612.
