# Correction: Aqueous microwave-assisted cross-coupling reactions applied to unprotected nucleosides

**DOI:** 10.3389/fchem.2026.1827612

**Published:** 2026-03-24

**Authors:** Frontiers Editorial Office

**Keywords:** nucleoside, nucleotide, Suzuki-Miyaura, Heck, water, green chemistry, microwave

In the published article, the **Conflict of Interest** statement lacked necessary details regarding a previous collaboration between the author Christophe Len and the reviewer Rafael Luque. An investigation by Frontiers’ Research Integrity auditing team found that this conflict of interest did not unduly impact the peer review process or scientific validity of the article. The correct statement appears below:

“The authors declare that the research was conducted in the absence of any commercial or financial relationships that could be construed as a potential conflict of interest. The author CL had previously collaborated with the reviewer RL.”

The original article has been updated.

